# Some Fallacies in the Treatment of Phenol (Carbolic Acid) Poisoning

**Published:** 1915-08

**Authors:** 


### SOME FALLACIES IN THE TREATMENT OF PHENOL (CARBOLIC ACID) POISONING.

---

The frequency with which individual chemical substances appear in the role of poisons varies from era to era. There was a period when arsenic, for example, occupied a more prominent place among the lists of fatalities by poisoning than it does at the present day. Acute intoxication with phenol (carbolic acid) has of late years assumed large proportions. The substance has become a popularly known household chemical which finds a diversity of applications. It is found at the bedside of the sick, in homes, in public places, in stables and in numerous similarly scattered places which indicate how readily accessible and how recklessly distributed the poison is. The comparatively recent period in which phenol has become thus "vulgarized" is indicated by the fact that it was not discovered until 1834, and its use for antiseptic purposes dates much later. Sometimes the poisoning has been due to medical misuse, though this is not very common; frequently it has been taken by mistake; many persons have employed it with suicidal intent. Under all these circumstances the seriousness of its improper use is enhanced by the fact that phenol is readily absorbed whether it be taken into the system by mouth, through the lungs in the form of a vapor or spray, through external application to the skin in dressings of wounds and otherwise, or even by absorption from the bowel when administered as an enema. The most significant feature of the toxic action of phenol is the rapidity with which the collapse and death may ensue.

Naturally the question of first aid and suitable antidotes is of pre-eminent interest in this connection. When coma and collapse have set in and reached the point at which artificial respiration becomes desirable, there is little prospect of resuscitation. Everything hinges on early procedure, particularly the removal of any unabsorbed poison, whether it be in the stomach or on the exterior surfaces. Dr. David I. Macht[1] of Baltimore has recently reviewed the current antidotal recommendations in detail before the Johns Hopkins Hospital Medical Society, devoting special attention to two drugs, alcohol and sodium sulphate, which have been used in this connection on seemingly rational grounds.

The internal use of alcohol in phenol poisoning originated, according to Macht, out of the demonstration that the destructive action of the phenol on the skin is promptly overcome by quickly rinsing it with strong alcohol.[2] A critical survey of the clinical reports, together with such experimental evidence as is available, indicates the uncertain basis on which the use of alcohol in phenol poisoning really rests. The same may be said of the treatment with sodium sulphate. Phenol is known to conjugate with sulphuric acid arising in metabolism and leave the organism in the form of an ethereal sulphate or sulphonate which appears to be comparatively harmless. Baumann and Preusse therefore suggested, years ago, that the administration of a sulphate in large quantities might act as an antidote against phenol. It is questionable, however, whether the latter combines with sulphates as such in the body. Sulphates have also been considered; but they are themselves too toxic to be taken internally.

Aside from the use of ordinary demulcents like white of egg, which has not proved of value, lime water and syrup of lime have been recommended in cases in which phenol has been swallowed, with the idea that they would form an insoluble compound in the stomach. Macht reports that they too are very unsatisfactory. According to his careful and extensive experiments on animals,

---

1 Macht, D. I.: An Experimental Study of Lavage in Acute Carbolic Acid Poisoning, Bull. Johns Hopkins Hosp., 1915, xxvi, 98.

2 Powell, S. D.: Med. Rec., New York, 1899, lv, 372. Buchanan, J. D.: ibid., lvi, 441.

death can often be averted by a thorough lavage of the stomach. In accord with the promptness of the onset of symptoms, the efficiency of lavage in phenol poisoning depends on the quantity of poison taken, on the time after poisoning that the lavage is begun, and on the solution used for washing the stomach. A strong solution of sodium sulphate appears to be most useful for this purpose, water coming next in efficiency. The favorable action of the sulphate in this connection is probably not a chemical one, but is perhaps due to some hindrance to absorption and possibly also to an induced purgation.

Macht has clearly demonstrated, contrary to the popular experience, that the internal use of alcohol in cases of phenol poisoning may be unfavorable. The conflicting opinions are reconciled, however, by his investigations. The influence of alcohol depends on the time of administration. If it is given after the ingestion of phenol, as must be the case therapeutically, the symptoms will be aggravated. The alcohol here acting as an excellent solvent for phenol, still further promotes its absorption. Death may actually be hastened. On the other hand, an animal previously intoxicated with alcohol can somehow withstand better the effects of phenol taken afterward. Macht suggests in accord with experience, that a previously drunken person will probably withstand the ravages of phenol better, whereas one that takes phenol mixed in alcohol or victims of phenol poisoning washed or doused with alcohol for therapeutic purposes, are more liable to perish. Clinical statistics are in agreement with this view. Taken together with the newer experimental evidence, they suggest, despite the advice found in some of the prominent textbooks on toxicology, that the use of alcohol in phenol poisoning should be strongly discouraged.—*Journal A. M. A.*, July 10, 1915.

